# Regulation Networks Driving Vasculogenic Mimicry in Solid Tumors

**DOI:** 10.3389/fonc.2019.01419

**Published:** 2020-01-14

**Authors:** Olga N. Hernández de la Cruz, José Sullivan López-González, Raúl García-Vázquez, Yarely M. Salinas-Vera, Marcos A. Muñiz-Lino, Dolores Aguilar-Cazares, César López-Camarillo, Ángeles Carlos-Reyes

**Affiliations:** ^1^Posgrado en Ciencias Genómicas, Universidad Autónoma de la Ciudad de México, Mexico, Mexico; ^2^Laboratorio de Cáncer de Pulmón, Instituto Nacional de Enfermedades Respiratorias “Ismael Cosío Villegas”, Mexico, Mexico; ^3^Laboratorio de Patología y Medicina Bucal, Universidad Autónoma Metropolitana Unidad Xochimilco, Mexico, Mexico

**Keywords:** cancer, vasculogenic mimicry, microRNAs, long non-coding RNAs, epithelial-mesenchymal transition, tumor microenvironment

## Abstract

Vasculogenic mimicry (VM) is a mechanism whereby cancer cells form microvascular structures similar to three-dimensional channels to provide nutrients and oxygen to tumors. Unlike angiogenesis, VM is characterized by the development of new patterned three-dimensional vascular-like structures independent of endothelial cells. This phenomenon has been observed in many types of highly aggressive solid tumors. The presence of VM has also been associated with increased resistance to chemotherapy, low survival, and poor prognosis. MicroRNAs (miRNAs) and long non-coding RNAs (lncRNAs) are non-coding RNAs that regulate gene expression at the post-transcriptional level through different pathways. In recent years, these tiny RNAs have been shown to be expressed aberrantly in different human malignancies, thus contributing to the hallmarks of cancer. In this context, miRNAs and lncRNAs can be excellent biomarkers for diagnosis, prognosis, and the prediction of response to therapy. In this review, we discuss the role that the tumor microenvironment and the epithelial-mesenchymal transition have in VM. We include an overview of the mechanisms of VM with examples of diverse types of tumors. Finally, we describe the regulation networks of lncRNAs-miRNAs and their clinical impact with the VM. Knowing the key genes that regulate and promote the development of VM in tumors with invasive, aggressive, and therapy-resistant phenotypes will facilitate the discovery of novel biomarker therapeutics against cancer as well as tools in the diagnosis and prognosis of patients.

## Introduction

Solid tumors can form new blood vessels through complex neovascularization mechanisms that include the following: (i) angiogenesis the development of new blood vessels by endothelial cells (ECs) from pre-existing vessels, (ii) vasculogenesis generated from EC precursors, (iii) intussusception the splitting of vessels through the insertion of tissue pillars, (iv) vessel co-option migration of tumor cells migrate along existing vasculature, (v) cancer stem cell (CSC) trans-differentiation whereby cancer cells trans-differentiate to ECs leading to the formation of blood vessels, and (vi) vasculogenic mimicry (VM) where the tumor cells mimic ECs and form blood vessel-like three-dimensional channels (1–3).

In particular, VM can enhance cancer cell migration, invasion, and metastasis as well as increased resistance to therapies. VM has been documented in diverse solid tumors such as breast cancer, liver cancer, ovarian cancer, gastric cancer, prostate cancer, and melanoma (4, 5). During tumorigenesis, the tumor microenvironment plays a vital role in the formation of the tumor vasculature. Deficient blood-vessel perfusion, hypoxia due to low oxygen pressure, and low-nutrient availability in the microenvironment lead to angiogenesis, metastasis, and tumor cell survival (6, 7). Hypoxia is a master regulator of various transcription factors and signaling pathways during of the development of VM in solid tumors (8). On the other hand, microRNA (miRNAs) and long non-coding RNAs (lncRNAs) regulate the expression of genes and signaling pathways in diverse tumor types, which contributes to the cancer hallmarks like metastasis via VM formation (9). Here, we summarize the latest advances in VM regulation in solid tumors. We first overview the role of the tumor microenvironment and the epithelial-mesenchymal transition (EMT) in VM from different types of tumors. We further described some mechanisms of VM. Finally, we detail the regulation by miRNAs and the regulation networks by lncRNAs-miRNAs as well as their clinical impact on VM.

## Role of Tumor Microenvironment and the EMT in the Development OF VM

The tumor microenvironment comprises the vasculature (blood vessels), extracellular matrix, stromal cells, immune cells, and signaling molecules. Poor blood-vessel perfusion in the tumor microenvironment is due to acidic pH, low nutrient levels, and hypoxia due to low oxygen pressure (10, 11). These aspects cause an imbalance in the angiogenic and anti-angiogenics factors that favor the invasion and metastasis of tumor cells. Tumor cell adaptation to the hypoxic microenvironment favors sustained angiogenesis (12). In particular, acidic pH and hypoxia are important factors for remodeling EMC. The acidic pH in solid tumors is due to the production of lactic acid during the fermentative metabolism produced by the high expression of Na+/H+ exchangers (NHE1), isoforms of anion exchangers, ${\text{Na/HCO}}_{3}^{-}$ co-transporters, H+/ATPases, carbonic anhydrase IX isoform, monocarboxylate transporters, and the vacuolar ATPase. The released proton (H+) acidifies the tumor microenvironment and diffuses toward the stroma increasing the tumor survival, proliferation, and angiogenesis (13).

A crucial event in the development, progression, and metastasis of malignant tumors is neovascularization. It supplies growth factors, nutrients, and oxygen that alter the vascularization of the tumor including sustained angiogenesis (3). A new mechanism of neovascularization is VM that leads to the formation of blood vessels by the tumor cells themselves independently of ECs. VM is characterized by the deregulation of genes such as vimentin, cadherins, and metalloproteases and can be detected by double PAS/CD31 staining (5, 14). Tumors that show VM are highly aggressive and metastatic invasive phenotypes that are resistant to therapies (15). VM is promoted by the hypoxic tumor microenvironment, acidic pH, low nutrient levels, and the EMT (16).

**Figure 1:**
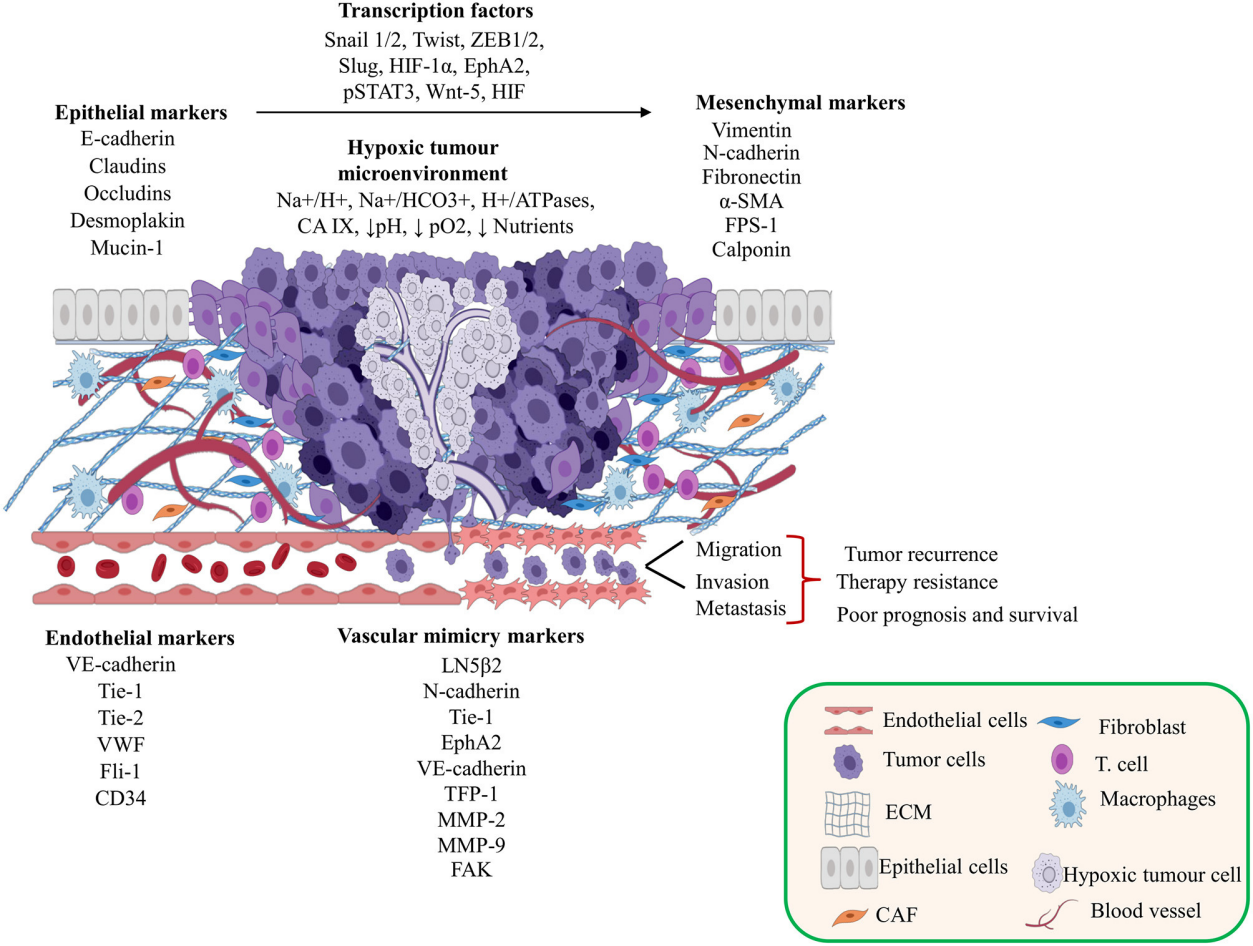
Contribution of the tumor microenvironment and epithelial-mesenchymal transition (EMT) in vasculogenic mimicry (VM) formation. This figure shows the tumor microenvironment effects and the relationships between transcriptional factors, EMT, and endothelial cell markers during the development of VM. Created with Biorender.com.

Several studies report that the hypoxic tumor microenvironment regulates different transcription factors mediated by HIF-1α. These factors induce VM development as well as the regulation of epithelial markers that favor the EMT in different solid tumors (Figure 1 and see Table 1) (16–42).

**Table 1:** Molecular factors that promote the EMT and VM in solid tumors.

**Factors promoting VM and EMT**	**Effects on cancer hallmarks and association with clinical parameters**	**Effects on proteins and signaling pathways associated with EMT-VM**	**Types of cancer**	**References**
Slug↑	Invasion, metastasis, TNM	↓ VE-cadherin, ↑vimentin	Hepatocellular, NSCLC	(17, 18)
Runx2↑	Migration, invasion	↓ VE-cadherin, ↑vimentin, ↑Galectin-3	Hepatocellular	(16)
pSTAT3	Migration, invasion, metastasis, TNM stage, poor prognosis	IL-6, ↓ VE-cadherin, ↑vimentin, ↑Twist1	Colorectal	(19)
CDK5	Migration, invasion	FAK/AKT	NSCLC	(20)
HIF-1α↑ HIF-2α↑	Shorter survival, poor tumor differentiation, late clinical stage, lymph node metastasis, poor prognosis	↓ E-cadherin, ↑vimentin, ↑Twist, ↑VE-cadherin, Claudin-4, ↑Slug, ↑MMP2, ↑MMP9, LOXL2, Twist1	Ovarian, colorectal, pancreatic, hepatocellular	(21–24)
ZEB1↑	TNM stage, lymph node, distant, migration	Src signaling, ↓ VE-cadherin, Flt-1	Prostate, colorectal	(25, 26)
ZEB2↑	Invasion, metastases	↑VE-cadherin, Flt-1, Flk-1, MMP	Hepatocellular	(27)
uPAR+	Metastasis, poor prognosis	↑Vimentin, ↑VE-cadherin; ↓ E-cadherin, ↑twist, ↑snail	Large-cell lung cancer	(28)
FZD2↑	Proliferation, apoptosis, migration, invasion	↓E-cadherin, ↑N-cadherin, ↑Vimentin, ↑Slug, ↑snail, Hippo signaling	Hepatocellular	(29)
Notch1↑	Invasiveness, poor prognosis	↑Hes1, ↓ E-cadherin, ↑vimentin, TGF-β1	Hepatocellular	(30)
DKK1↑	Migration, invasion, proliferation, aggressive, poor prognosis.	↓E-cadherin, ↑N-cadherin, ↑Vimentin, ↑MMP2, ↑MMP9, ↑β-catenin-nu	NSCLC	(31)
Netrin-1	Migration, invasion	↓E-cadherin, ↑N-cadherin, PI3K/AKT and ERK	NSCLC	(32)
EphA2↑	Migration, invasion	↑Vimentin, ↑twist	Head and neck squamous cell	(33)
LGIR1	Migration, invasion	↓E-cadherin, ↑N-cadherin, EGFR/ERK	Melanoma	(34)
MIF↑	High-grade tumor, poor survival	CXCR4/AKT/EMT	Glioblastoma	(35)
Twist1	Migration, invasion, metastasis	↓E-cadherin, ↑N-cadherin, MMP9, Bcl-2	Hepatocellular	(36, 37)
MACC1↑	Poor prognosis	HGF/c-Met-↑TWIST1/2	Gastric	(38)
Wnt5a	Metastasis	PKCα, ↓ E-cadherin, ↑vimentin	Ovarian	(39)
HMGA2	TNM stage, metastasis, recurrence	↑VE-cadherin, ↑twist1	Gastric	(40)
ROCK1	Invasion, metastasis	↓ E-cadherin, ↑vimentin,RhoA/ROCK	Hepatocellular	(41)
Sema4D	Migration	RhoA/ROCK	NSCLC	(42)
LOXL2	Metastasis, poor prognosis	↓ E-cadherin, ↑vimentin	Hepatocellular	(23)

*↑ High expression, ↓ low expression. EMT, epithelial-mesenchymal transition; VM, vascular mimicry; NSCLC, non-small cell lung cancer*.

We show some reports of the role of the tumor microenvironment and EMT in the VM. In melanoma, hypoxia activates MMP-2 and MMP-9 expression promoting invasion to adjacent tissue. There is also deficient blood perfusion due to HIF-1α high expression (43). In addition, an increase in HIF-1α causes high expression of VEGF that facilitates the formation of VM. In a different study, the authors found that melanoma cells showed an increase in Bcl-2 expression promoting the formation of three-dimensional tubular structures via the VE-cadherin up-regulation mediated by Bcl-2 (44).

Ovarian cancer includes high expression levels of human chorionic gonadotropin and HIF-1α, which contribute to cell proliferation and tumor growth. They also lead to VM via the luteinizing hormone receptor (45–47). Only 25% of ovarian cancer biopsies are positive for VM, which correlates with hypoxia and EMT and is due to the high expression levels of HIF-1α, vimentin, VE-cadherin, Twist1, and Slug (21). These factors decrease E-cadherin expression. A hypoxic environment in breast cancer can increase the levels of HIF-1α, VE-cadherin, MMP-9, Cdc42, EGFR, p-Akt, and p-mTOR to promote the development of VM via the HIF-1α/VE-cadherin/MMP-9, MMP-2 signaling pathway (48). In the colorectal cancer HCT-116 cell line, hypoxia-induced development of VM is due to an increase in the zinc finger E-box binding homeobox 1 (ZEB1) and HIF-1α as well as with high vimentin expression and loss of E-cadherin expression in EMT (25).

The relationship between Bcl-2/Twist1 and Bcl-2-Bmi1 promotes EMT and development of VM through loss expression of E-cadherin and increased vimentin expression in hepatic cancer cells. VM is also caused by the translocation of Twist1 to the nucleus via Bcl-2 due to hypoxia (49–51). In biopsies of hepatic cancer, high expression levels of Notch1 and Hes1 were associated with VM. In hepatic cancer HepG2 and MHCC97-H cell lines, the Notch1 expression was higher in HepG2 favoring invasiveness by inducing the EMT through an increase in vimentin and loss of E-cadherin expression. These events are mediated by stimulation of TGF-β1 (30).

Glioma biopsies have increased expression in HIF-1α, MIF, and CXCR4. This has been observed in hypoxic regions of the tumor and is associated with VM development. In U87 and U251 glioma cell lines, high expression of MIF and CXCR4 promoted EMT and VM formation. In *in vivo* assays, MIF induces VM through the CXCR4-AKT-signaling pathway (35). In other reports, the glioma cell line SHG-44 transfected with pEGFP-Cl-LRIG1 and overexpressing LRIG1 inhibits VM promoted by hypoxia as well as migration, invasion, and proliferation. In addition, LRIG1 expression repressed signaling of the EGFR/PI3K/AKT and EMT through an increase in E-cadherin and low vimentin expressions (52).

In melanoma, LRIG1 shows the same effects as glioma, but these are mediated by blocking via EGFR/ERK signaling (34). SACC-83 and SACC-LM salivary adenoid cystic carcinoma cell lines (SACC cells) have VM due to growth factors such as VEGFA that promote the development of VM mediated by hypoxia favoring migration and invasion as well as the EMT. Furthermore, the self-renewal capacity of SAAC-LM cells was due to the acquisition of the stem cell phenotype through VEGFA over-expression as well as an increase in the expression of N-cadherin, vimentin, CD44, and ALDH1 and loss of E-cadherin. These authors reported that only 26.3% of biopsies showed channel formation typical of VM. This phenomenon is related to HIF-1α and VEGFA expression (53). High expression levels of signal transducer and activator of transcription-3 (STAT3), p-STAT3, and HIF-1α in gastric cancer tissues for positive VM were associated with metastasis, degree of differentiation, and prognosis (54). On the other hand, esophageal squamous cancer cell lines Eca 109 and TE13 increased HIF-1α expression in hypoxic microenvironments. This promoted the VE-cadherin expression and led to VM development through the regulation of EphA2 and laminin subunit 5 gamma-2 (LN5γ2) expressions (55).

The EMT promotes the VM induced by hypoxia through the regulation of different transcriptional factors that promote the most aggressive, invasive, and metastatic phenotypes. These phenotypes are frequently therapy resistant with high recurrence.

## Mechanisms of VM in Human Cancers

**Figure 2:**
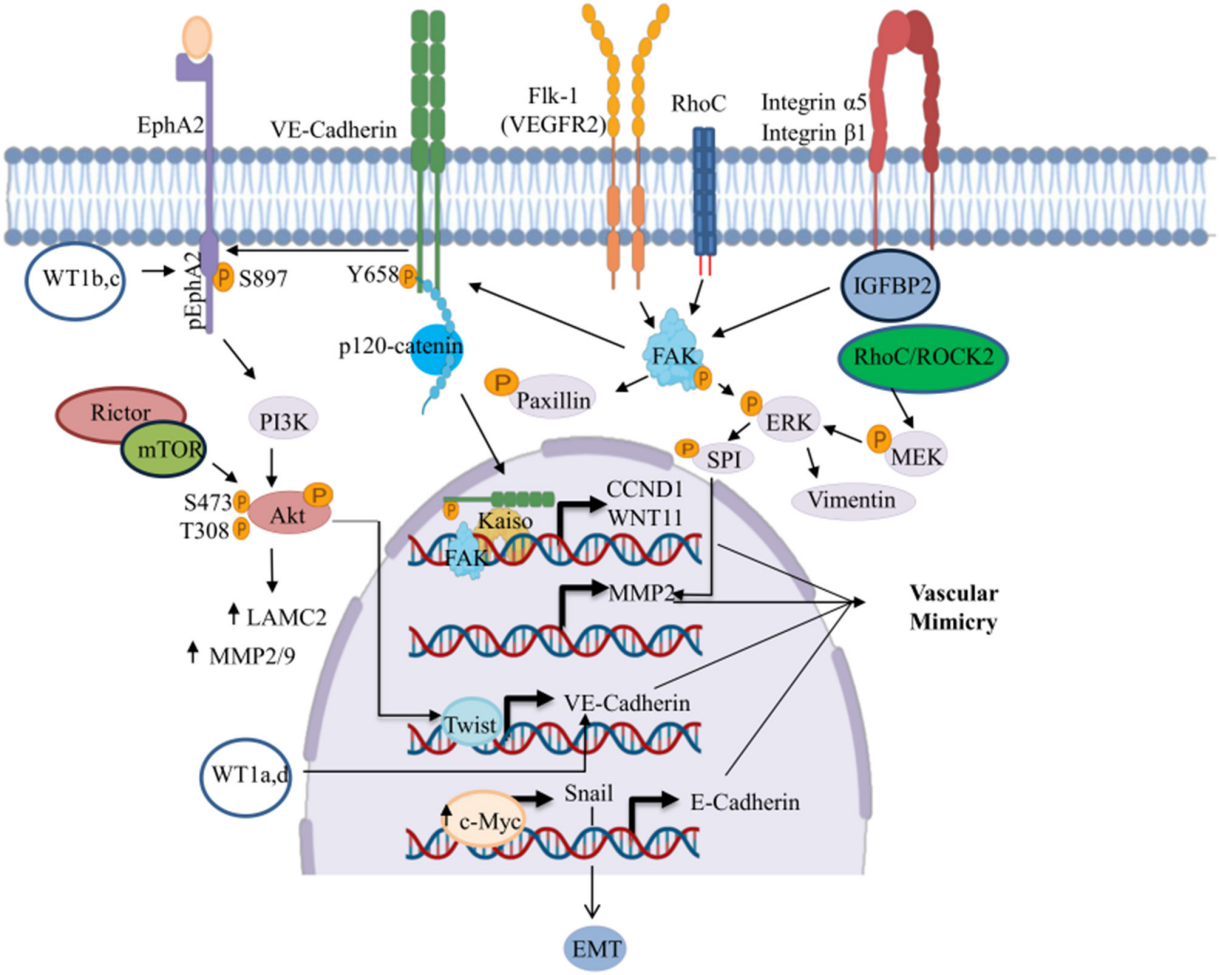
Signaling pathways involved in the regulation of VM. This figure shows the signaling pathways triggering the transcriptional activation of genes involved in the development of VM. ↑, Increased; 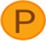, phosphorylated. Created with Biorender.com.

Many studies have reported the participation of several transcription factors impacting diverse signaling pathways including EphA2, VE-cadherin, VEGFR2 (Flk-1), Rho, and integrins. These pathways regulate the VM development (Figure 2). In this review, we summarize some mechanisms related to the development of VM in solid tumors. Some of these are driven by different receptors like the Eph receptor tyrosine kinases and the ephrin ligands that have been extensively implicated in carcinogenesis (56).

EphA1 and EphA2 are the most well-characterized molecules in solid tumors. They are implicated in VM formation and angiogenesis. High invasive MUM-2B melanoma cell line develops vascular pattern networks that are PAS positive. Such networks are associated with high expression and phosphorylation of EphA2 (57). Recent studies *in vivo* and *in vitro* in prostate cancer showed that VM development was associated with high expression of EphA2 and PI3K. PI3K/AKT regulates the activity of EphA2 through phosphorylation at Ser897 position (58). In addition, Yeo and coworkers showed that fetal bovine serum at 1–5% induced VM formation in prostate cancer PC-3 cells as well as phosphorylation of EphA2. This also increased the expression of VE-cadherin and Twist and activated AKT signaling. These changes were accompanied by an increase in MMP-2 and LN5γ2 protein levels (59). More experiments are needed to analyze whether cellular stress caused by reduced concentrations of fetal calf serum induced the development of VM.

Liang and coworkers found that Rictor, a component of mTOR2 signaling highly expressed in melanoma, was related to the presence of VM. These were associated with poor patient survival. The authors showed that VM was regulated through an increased activity of AKT-MMP-2/9 signaling and phosphorylation of AKT at Ser473 and Thr308 mediated by Rictor (60). In breast cancer, overexpression of B and C isoforms of WT1 promoted VM development due to the EphA2/β-catenin/vimentin signaling pathway (61). In contrast, highly aggressive gallbladder carcinomas develop VM mediated by high levels of MMP-2 and MT1-MMP the overexpression of EphA2, FAK/PI3K, and LN-5γ2 signaling pathways, and via Paxillin-P signaling *in vitro* and *in vivo* (62).

Another mechanism for promotion of VM is mediated by VE-cadherin, one of the major endothelial adhesion molecule controlling intercellular junctions for blood vessel formation. This occurs via vascular endothelial growth factor receptor (VEGFR) function (63). Delgado-Bellido and coworkers found that malignant melanoma cells show constitutive expression of phosphorylated VE-cadherin at the Y658 position and forms a complex with p120-catenin and the transcriptional repressor kaiso. The high expression of nuclear phosphorylated VE-cadherin activated kaiso-dependent genes CCDN1 and WNT11 that increase the VM formation (64). Other study in melanoma showed that the c-Myc proto-oncogene was highly expressed in metastatic melanoma and induced VM through Snail activation inducing EMT by TGF-β/Snail/E-cadherin signaling pathways. c-Myc increases Bax expression causing a decrease in the Bcl2/Bax ratio through bilinearly patterned programmed cell necrosis. Necrosis forms empty spaces similar to blood vessels that serve as a support for VM formation under severe hypoxia (65). On the other hand, HER2-positive tumors showed high VM formation via VE-cadherin regulation (66).

Integrins are cellular adhesion receptors for cell attachment to the extracellular matrix. They transmit signals between cells and microenvironment. Integrins have multiple functions in cancer from initiation through metastasis, and they have been implicated in VM in various cancer types (67). In glioblastoma, Liu and coworkers analyzed tumors with VM and found a positive correlation between high levels of insulin-like growth factor–binding protein 2 (IGFBP2) and CD144/MMP-2 expression. They further found that overexpression of IGFBP2 increased tubule network formation through activation of CD144 and MMP-2, which was mediated by the binding of IGFBP2 to integrin α5/β1 and activation of the FAK/ERK/SPI pathway (68). Another report in glioblastoma multiforme showed that the presence of tumor-associated macrophages with M2 phenotype 2 infiltrating the VM-positive tissue area was associated with high levels of cyclooxygenase-2 (COX-2). Co-cultures of the U87 cell line with M2 macrophages activated by interleukin-4 promoted VM development through the prostaglandin E2/EP1/PKC pathway with high COX-2 and α-SMA expression and low VE-cadherin expression (69). In contrast, breast cancer cell lines that overexpress COX-2 vascular channels were also reported; this event was inhibited by COX inhibition (celecoxib) or siRNAs and was restored upon addition of exogenous prostaglandin E2 (70).

VEGF signaling via tyrosine kinase receptor VEGF receptor 2 (Flk-1) has a critical role in tumor angiogenesis and promotion of VM in cancer (71, 72). Blood vessels detected in the VM of glioblastoma cells are integrated by mural cell-lined vasculature. In glioblastoma, U87 and GDSC cell lines promoted VM formation. This formation was mediated by high expression of Flk-1 and VE-cadherin. Suppression of Flk-1 activity with SU1498 inhibitors in turn inhibits VM formation as well as FAK and ERK1/2 signaling pathways (73).

RhoC (Ras homolog gene family member C) regulates cytoskeletal organization and affects the motility of cancer cells favoring invasion and metastasis as well as progression and VM formation (74). In hepatocellular carcinoma (HCC), vascular channels were formed, and these vessels were associated with RhoC FAK/Paxillin signaling regulation as well as with high expression of RhoC/ROCK2, VE-cadherin, and MMP2 mediated by ERK/MMPs signaling (75).

## CSCs and VM

CSCs are a subgroup of tumor cells that are multipotent with the capacity for self-renewal and differentiation as well as phenotypic and functional features of stem cells. In recent years, several reports have shown the role of CSC in the development of VM; they can form vascular-like structures that mimic embryonic vascular network patterns that are pivotal in tumor progression (76). For instance, in colorectal cancer, the Wnt/β-catenin pathway can induce VM formation through high Wnt3 and low β-catenin expressions. This pathway can also increase the expression of VEGFR2 and VE-cadherin. Thus, the Wnt/β-catenin activation favors the acquisition of endothelial phenotypes (77).

The glioblastomas undergo trans-differentiation into CD133-positive vascular ECs that promote the formation of glioma stem-like cells that can initiate the development of VM through VEGFR2 and VE-cadherin (72). In HCC, the Frizzled-2 gene (FZD2) induces proliferation, migration, and invasion due to its high expression. HCC also has decreased E-cadherin expression and increased N-cadherin, Snail, and Slug expressions. This promotes the EMT and VM formation. FZD2 also regulates the transcription factors Nanog and SOX2 in pluripotent cells favoring stemness. Moreover, the enrichment analysis of DEGs showed that FZD2 has a close relationship to the Hippo pathway mediated by the activity of YAP and TAZ (29). The tumorigenic growth of melanoma is shaped by stem-like cells. This cancer can grow to form spheroid cells that generate laminin networks similar to VM. These laminin networks have high expression of VE-cadherin, VEGFR-1, and nestin stem cell-associated biomarkers (78). These reports demonstrate that subpopulations of CSCs can transdifferentiate, and they contribute to development of VM in solid tumors.

## Regulation OF VM by miRNAs in Solid Tumors

miRNAs are post-transcriptional regulators of gene expression; they participate in degradation and/or inhibition of translation of their target genes (79). The alteration of the expression of multiple miRNAs has been reported in diverse types of solid tumors, and they act as oncogenes or tumor suppressors (80). Dysregulation of both groups correlates with diverse biological processes such as proliferation, invasion, migration, and VM in human cancer (see Table 2) (81–102). An important signaling pathway is VE-cadherin; it is one of the first factors identified as a regulator of VM. Liu and coworkers recently demonstrated the low expression of miR-27b in ovarian cancer cells. Restoring miR-27b expression in ovarian Hey1B and ES2 cancer cell lines significantly decreased intracellular VE-cadherin expression. This inhibits invasion, metastasis, and VM caused by the direct binding of miR-27b to the 3′-UTR region of VE-cadherin (89). In HCC cell lines, the miR-27a-3p and miR-17 were down-regulated and associated with high Bcl-2 expression. The VE-cadherin, MMP-2, Twist1, HIF-1α, and VEGFA also lead to VM. In hepatic tumors, these genes were associated with poor prognosis of patients. Moreover, miR-27a-3p functions as a tumor suppressor for invasion and metastasis and is mediated by downregulation of VE-cadherin and EMT (96, 97). The miRNAs also regulate the EMT and facilitate the development of capillary-like structures in the tumors. They adopt invasive and metastatic properties. There was a decrease in miR-186 expression levels in P69 and M12 prostate cancer cell lines and tissues of patients with metastatic prostate cancer. The restoration of miR-186 suppresses cell motility, invasion, colony formation, and three-dimensional culture growth, and inhibits the EMT through the negative modulation of Twist1 transcription factor (100). miR-124 in cervical cancer induced the suppression of the EMT process and decreased migration, invasion, and VM due to its specific interaction with 3′UTR of the angiomotin like 1 (AmotL1) protein that regulates cell migration related to angiomotin (87, 102).

**Table 2:** Modulation of VM by microRNAs (miRNAs).

**miRNAs**	**Targets**	**Biological function**	**Cancer type**	**References**
mir-200C, mir-183, mir-96, mir-182 mir-299-5P mir-125a, Let-7e mir-193b miR-204	ZEB-1 OPN IL-6, IL-6R,STAT3 DDAH1 PI3K, c-SRC, FAK	Inhibit VM, increased proliferation, chemotherapy, poor prognosis	Breast	(82) (83) (84) (85) (86)
mir-124	AmotL1, STAT3	Inhibit migration, invasion, EMT, VM	Cervical, oral	(87, 102)
mir-200a mir-27b miR-765	EphA2 VE-cadherin VEGFA, AKT1, SRC-α	Poor survival, inhibit VM, Inhibit migration, invasion	Ovarian	(88) (89) (90)
miR-9 mir-26b miR-Let-7f mir-584-3p miR-141	STMN1 EphA2 POSTN ROCK1 EphA2	Decreased tumor growth, VM, glioma grades II, III and IV, prognostic indicator, proliferation, migration, invasion	Glioma	(91) (92) (93) (94) (95)
miR-27a miR-17 miR-27a-3p mir-101 mir-29b	CDH5, SMAD2, TGFBR1, VEGF VEGF, HIF1A, MMP2 VE-cadherin TGF-BR1,Smad2 SDF1 IL-6, STAT3, MMP-2	Poor prognosis, represses early metastasis, EMT, VM	Hepatocellular	(96) (97) (98) (99)
miR-186	TWIST1	Inhibit tumor progression, invasion, colony formation, EMT	Prostate	(100)
miR-490-3p	TR4, Vimentin	Promote metastasis, VM	ccRCC	(81)
miR-409-3p	ANG	Suppresses proliferation, tumor growth, metastasis, VM	Fibrosarcoma	(101)

Several reports have shown that Eph2A expression is regulated by different miRNAs. In glioma and glioblastomas cell lines, overexpression of miR-26b and miR-141 inhibited VM formation through their specific binding with the Eph2A 3′-UTR region. Also, both miRNAs regulate cell proliferation, migration, and invasion (92, 95). Ovarian cancer has a decrease in the expression levels of miR-200a that induce VM development. The restoration of miR-200a inhibits VM through modulation of the expression of EphA2. Also, low miR-200a expression has been associated with tumor grade and metastasis (88).

VEGF expression has been detected in hypoxic environments, and its secretion by tumor cells plays a crucial role in the tumor angiogenesis and VM formation (71, 72). Salinas-Vera et al. reported that miR-765 decreased VEGF expression after hypoxic conditions in ovarian cancer. Restoration in SKOV3 cells resulted in a significant inhibition of VM suggesting that miR-765 coordinates VM formation through modulation of the VEGFA/AKT1/SRC-α signaling pathway (90). The same group reported that miR-204 reduced the expression and phosphorylation of 13 proteins involved in the PI3K/AKT, RAF1/MAPK, VEGF, and FAK/SRC signaling pathways in MDA-MB-231 breast cancer cell line. These pathways impact VM development; its restoration repressed the VM formation and regulated the PI3K/AKT signaling pathway through its specific interaction with PI3K and SRC (86).

Multicellular spheroids derived from MCF-7, MCF10AT, and MCF10DCIS cell lines reproduce the architecture and tumor physiology observed *in vivo*. These models show high expression of osteopontin (OPN) oncoprotein via downregulation of miR-299-5p leading to vascular structures similar to VM (83). However, more studies are required in other type of tumors to correlate whether multicellular spheroids are linked with VM.

In gliomas, the expression decrease of miR-584-3p has been related with VM formation. The restoration of this miRNA expression inhibits VM formation *in vitro* through direct binding with the 3′-UTR region of ROCK1 (94). In this same tumor, miR-Let-7f overexpression suppresses VM by repression of periostin (POSTN) that can induce migration of the cells. Overexpression of miR-9 a tissue-specific miRNA in the central nervous system increases apoptosis, suppresses tumor volume, and decreases cell proliferation and migration as well as VM formation *in vivo* and *in vitro* through negative regulation of the oncoprotein Stathmin (STMN1) (91, 93).

These studies show the essential role of the miRNAs in regulating the VM, in addition to other signaling pathways related to hallmarks cancer as cell proliferation, invasion, migration, and sustained angiogenesis in which new microcirculation process are implicated in therapy resistance and tumor recurrence.

## lncRNAs-miRNAs-mRNAs Regulation Networks of the VM and Their Clinical Relationship

**Figure 3:**
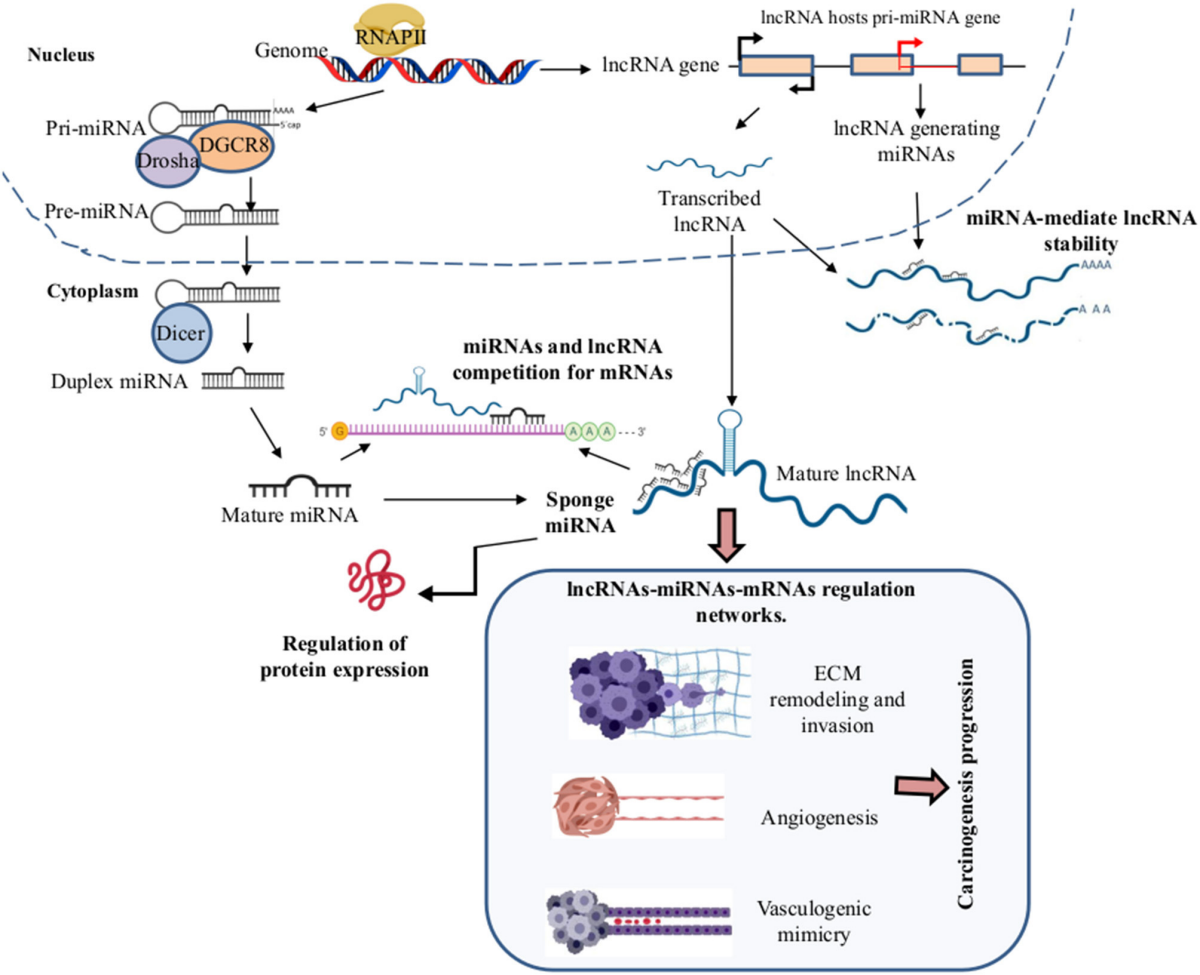
Long non-coding RNAs (lncRNAlncRNAs)–microRNAs (miRNAs)–mRNAs regulation networks in the VM development. During the biogenesis of the miRNAs and lncRNAs in the nucleus by the RNAPII, the pri-miRNA and lncRNAs are exported to the cytoplasm where the mature miRNAs and lncRNAs are formed, respectively. In the cytoplasm, LncRNAs can function as sponges of miRNAs by competition for the binding to mRNA target genes, leading to the formation of complex regulatory networks of lncRNAs-miRNAs-mRNAs, promoting angiogenesis, extracellular matrix (ECM) remodeling, invasion, migration, EMT, metastasis, and VM formation in solid tumors. Created with Biorender.com.

**Figure 4:**
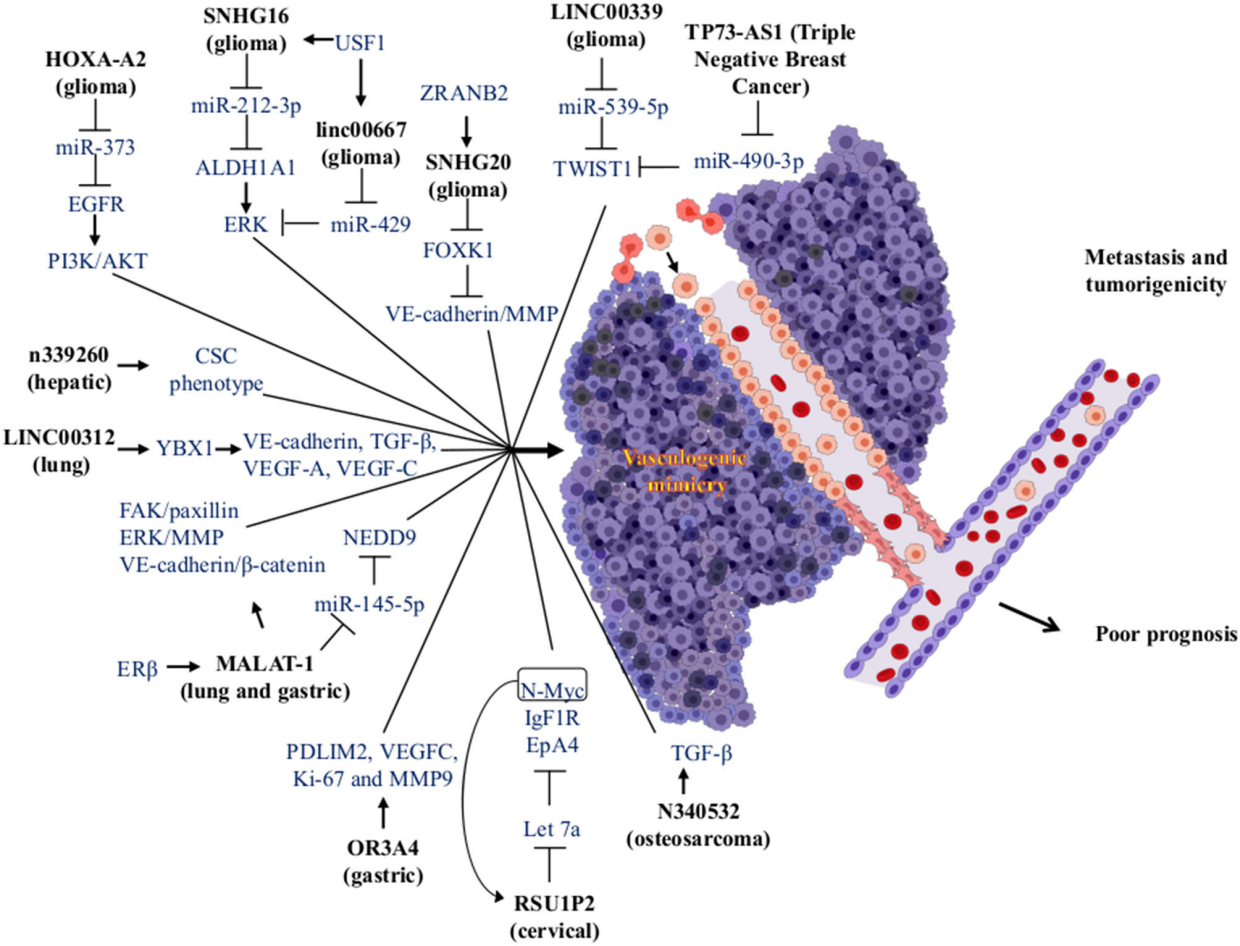
Modulation of miRNAs and lncRNAs associated with VM formation. The lncRNAs (bold letters) can interact with miRNAs or proteins, impacting various signaling pathways, which in turn trigger the formation of VM structures in tumors. The VM has been associated with an increase in metastasis and tumorigenicity, being factors that cause a poor prognosis in cancer patients. Created with Biorender.com.

lncRNAs are a heterogeneous group of RNA molecules longer than 200 nucleotides. They have a dynamic role in the transcriptional and translational regulation of key genes in several diseases including of cancer. Their aberrant lncRNAs expression in the tumorigenesis contributes to metastasis, progression, and patient survival, as well as with VM development (103). The lncRNAs act as a competitive endogenous RNA. They change the expression transcriptional by competing for specific miRNAs binding sites altering their interaction. The regulation of lncRNAs in the miRNAs forms a complex regulatory network of lncRNAs-miRNAs-mRNAs (104, 105). This network promotes the acquisition of cellular phenotypes such as migration, invasion, angiogenesis, and VM (Figure 3). lcRNAs are involved in a wide range of cellular processes regulating gene expression through multiple molecular mechanisms such as mRNA degradation and regulation of protein activity; scaffolds in the assembly of complexes, guides, decoys, or riborepressors; riboactivators; translational inhibition; chromatin remodeling; and miRNAs sponging (106). LncRNAs decrease miRNA target concentration within the cell through of their specific binding, which inhibits their function. Interestingly, hypoxia induces the expression of many lncRNAs and, similarly to miRNAs, lncRNAs are differentially expressed in diverse tumors leading to cancer hallmarks like metastasis via VM formation (107) (Figure 4).

For instance, Guo et al. compared the differential expression of lncRNAs in metastatic tissue, primary gastric cancer, and normal gastric tissue. High expression of the lncRNA olfactory receptor [family 3, subfamily A, member 4 (OR3A4)] is associated with gastric cancer metastasis, but in gastric cancer, this is related to clinicopathological features. This lncRNAs promotes cell proliferation, migration, and invasion in gastric cancer. The *in vitro* assays of OR3A4 cells induced VM and tubule formation in HUVECs cells. The high expression in cell lines induced angiogenesis in chicken embryos mediated by VEGF-C and MMP-9. This promotes the activation of target genes PDLIM2, MACC1, NTN4, and GNB2L1. Furthermore, high expression of OR3A4 has been observed in different types of cancer like esophageal, gastric, colon, gallbladder, pancreatic, hepatic, and several gastric cancer cell lines (SNU-16, AGS, SNU-1, KATOIII, MKN45, NCI-N87, and SGC7901) and one immortalized gastric mucosa cell line (GES-1) (108). In another report, gastric cancer was detected via the overexpression of the lncRNA metastasis-associated lung adenocarcinoma transcript 1 (MALAT-1). MALAT-1 is associated with poor prognosis, endothelial vessels formation, and VM. Its overexpression increased migration, invasion, vascular permeability, and tumorigenicity in a nude mice model. Also, MALAT-1 regulates angiogenesis and VM through modulation of VE-cadherin/β-catenin complex, ERK/MMP, and FAK/Paxillin signaling pathways (109).

In cervical cancer, high expression of the lncRNA Ras suppressor protein 1 pseudogene 2 (RSU1P2) was associated with VM formation. Its overexpression reduces apoptosis, cell cycle progression, and the EMT in nude mice-induced tumorigenesis. This increases the cell viability, proliferation, migration, invasion, and VM. The lncRNA and miR-let-7a compete for binding sites for IGF1R, N-myc, and EphA4, which inhibits their suppressive effect. Interestingly, high expression of N-myc induced the overexpression of RSU1P2 and decreased the expression of let-7a forming a positive feedback loop (110).

In glioma tissues and glioma cell lines, the lncRNA LINC00339 was correlated with VM formation by its up-regulation. Knockdown of LINC00339 decreases cell proliferation, migration, and invasion, and led to the development of VM. There is also increased survival via reduction of tumor growth through interactions with miR-539-5p. This system in turn increased the expression of TWIST1 that binds to promoters of MMP-2 and MMP-14 stimulating its transcription (111). Furthermore, the lncRNAHOXA cluster antisense RNA 2 (HOXA-AS2) was overexpressed in tissues and cell lines of glioma correlating with cell viability, migration, invasion, and VM formation via negative regulation of miR-373 and EGFR over-expression. EGFR enhances the expression levels of VE-cadherin as well as the activity of MMP-2 and MMP-9 metalloproteases in U87 and U251 cell lines. This favors VM development through activation of the PI3K/AKT signaling pathway (112).

Another report in gliomas found high expression of upstream transcription factor 1 and aldehyde dehydrogenase-1. These promoted cell proliferation, migration, invasion, and VM development. These molecules were related to histopathological grading. This kind of tumor has high expression of lncRNAs SNHG16 and linc00667 that induce the VM regulating of upstream transcription factor 1 and aldehyde dehydrogenase-1 targets, which are regulated by miR-212-3p and miR-429 and have low expression in gliomas. The inhibition of SNHG16 and linc00667 caused overexpression of miR-212-3p and miR-429 and the inhibition of VM (113).

In lung cancer, the estrogen receptor β interacts with different estrogen response elements located in the lncRNA MALAT-1 promoter to increase the expression. Upregulation of MALAT-1 decreases the expression of miR-145-5p and increases overexpression of the NEDD oncogene (a target of miR-145-5p and linked to non-small cell lung cancer metastasis). This promotes VM formation and cell invasion *in vitro* and *in vivo* (114).

Li et al. reported the high expression of the lncRNA small nucleolar RNA host gene 7 (SNHG7) in colorectal cancer. SNHG7 is involved in cancer progression with poor prognosis and increased cell proliferation (*in vitro* and *in vivo*), cell cycle progression, migration, invasion, and VM formation. It blocks apoptosis in cell lines. They showed that miR-34a is a direct target of SNHG7. It regulates the expression of the GalNAc transferase 7 (GALNT7). Thus, SNHG7 could increase the expression level of GALNT7 oncogene by sponging miR-34a. They also demonstrated that SNHG7, miR-34a, and GALNT7 can increase the activity of the PI3K/AKT/mTOR pathway in colorectal cancer cell lines with different metastatic degrees (115). In triple-negative breast cancer, the high lncRNAs expression TP73 antisense RNA 1 (TP73-AS1) was associated with VM. Besides, TP73-AS1 was overexpressed in MDA-MB-231 cells and binds specifically to miR-490-3p. Furthermore, miR-490-3p is regulated by TP73-AS1 inducing an increase in the TWIST1 (target of miR-490-3p) expression and promoting the development VM (116).

LncRNAs are regulated by various RNA-binding proteins. Li et al. demonstrated that the zinc-finger RAN-binding domain-containing protein 2 (ZRANB2) is one RNA-binding protein that is overexpressed in tissues and cell lines of glioma. Due to protein-RNA interactions, ZRANB2 stabilizes the SNHG20 lncRNA, promotes the degradation of Forkhead box K1 (FOXK1), and increases the transcription of MMP-1, MMP-9, and VE-cadherin; this stimulates proliferation, migration, invasion, and VM development in this type of cancer (117).

In HCC, high expression of n339260 lncRNA was related to the expression of stem cell markers (c-myc, sox2, and Nanog) as well as with high expression of VE-cadherin, VM formation, metastasis, poor prognosis, and low survival of the patients. Therefore, this lncRNA can induce VM through a CSC phenotype (118).

Another lncRNA related to the development of VM in lung cancer is LINC00312. Its high expression was observed in metastatic lung adenocarcinoma patients and is associated with poor survival. Overexpression of LINC00312 in mice increase the number of metastatic tumor nodules by increasing migration, invasion, and stimulation of VM. LINC00312 mediates the aforementioned effects through direct binding to the transcription factor Y-Box Binding Protein 1 (YBX1), which induces the expression of angiogenic genes such as VE-cadherin, TGF-β, VEGF-A, and VEGF-C (119).

In osteosarcoma, Ren et al. reported that lncRNAs and mRNAs are differentially expressed and are associated with VM in the extremely aggressive 143B osteosarcoma cell line. They found that lncRNA n340532 that is silenced in 143B cells by siRNA reduces the VM formation *in vitro*. Nude mice were injected with n340532-knockdown in 143B cells and develop smaller tumors with fewer metastatic nodules and VM channels vs. control mice (120).

These few studies show that lncRNAs-miRNAs-mRNAs play critical roles in the regulation of VM development. They have clinical implications in several types of highly aggressive cancer because they are involved with tumor progression, poor survival and prognosis, resistance to therapy, and tumor recurrence. Thus, some miRNAs and lncRNAs have been proposed as prognostic and diagnostic biomarkers in solid tumors, although the mechanisms of interaction between lncRNAs-miRNAs-mRNAs have not been completely elucidated yet. A study of these interactions can better explain these regulatory networks and can explain how the cell coordinates complex events during VM. The data can also better explain the clinicopathological relationships in the development of tumors.

## Conclusion and Perspectives

This review provides the most recent evidence on the impact of VM as an alternative way of generating blood vessels in tumors. The tumor microenvironment exerts a clonal selection pressure on the tumor cells to adapt to the microenvironment with low oxygen pressure and acidic/hypoxic environments. These changes promote the formation of VM in solid tumors where HIF-1α is the protagonist modulating different molecules such as VE-cadherin, EphA2, LN5γ2, MMPs, VEGF, STAT3, Bcl-2, and signaling pathways as TGF-β1, EGFR/PI3K/AKT, and RhoA/ROC. With all these antecedents, HIF-1α is considered a master regulator that promotes MV.

During EMT, the structure of the cytoskeleton of the tumor cell undergoes changes that contribute to the plasticity of the tumor; it mimics the characteristics of ECs, which help migration, invasion, and metastasis of cells. In this context, it is necessary to understand the mechanism of the EMT process and the relationship that exists with the VM because these events provide properties to the tumor cells that make the anti-angiogenic therapies inefficient. In addition, we can identify therapeutic targets that contribute to the treatment of the most frequent solid tumors that usually respond to the start of therapy and subsequently relapse and do not respond to treatment. Examples include small cell lung cancer, pancreatic, osteosarcomas, melanomas, and sarcomas. Different miRNAs and lncRNAs may inhibit VM; they could be critical to the design of new therapeutic strategies. Nevertheless, the contribution of lncRNAs in VM remains largely unknown, few miRNAs and lncRNAs have been functionally studied in detail, and many important questions remain to be addressed. With the development of new and powerful genomic technologies, particularly next-generation sequencing, several lncRNAs can be identified in the future. These would be associated with different cancer types for their use in clinical practice.

## Author Contributions

ÁC-R, OH, and JL-G organized the entire manuscript, wrote the draft, and revised the last version of manuscript. ÁC-R, RG-V, and CL-C wrote the Mechanisms of VM in Human Cancers and CSCs and VM sections. YS-V, DA-C, and MM-L wrote the Role of Tumor Microenvironment and the EMT in the Development of VM section. MM-L, JL-G, and DA-C wrote the section on the role of EMT in VM and wrote the section on the role of miRNAs and signaling pathway in vasculogenic mimicry in solid tumors and modulation of EMT-VM by miRNAs. YS-V, RG-V, CL-C, OH, JL-G, and CL-C wrote the section on the MiRNAs and lncRNAs and the regulation networks by lncRNAs-miRNAs and their clinical relation to the VM. Figures 1–4 and Tables 1, 2 were designed and made by ÁC-R, MM-L, YS-V, and OH.

### Conflict of Interest

The authors declare that the research was conducted in the absence of any commercial or financial relationships that could be construed as a potential conflict of interest.
